# Smoking Cessation and Vaping Cessation Attempts among Cigarette Smokers and E-Cigarette Users in Central and Eastern Europe

**DOI:** 10.3390/ijerph17010028

**Published:** 2019-12-18

**Authors:** Mateusz Jankowski, Joshua Allan Lawson, Andrei Shpakou, Michał Poznański, Tadeusz Maria Zielonka, Ludmila Klimatckaia, Yelena Loginovich, Marta Rachel, Justína Gereová, Łukasz Minarowski, Ihar Naumau, Kamil Kornicki, Paulina Pepłowska, Valeriy Kovalevskiy, Asta Raskiliene, Krzysztof Bielewicz, Zuzana Krištúfková, Robert Mróz, Paulina Majek, Szymon Skoczyński, Jan Eugeniusz Zejda, Grzegorz Marek Brożek

**Affiliations:** 1 Department of Epidemiology, School of Medicine in Katowice, Medical University of Silesia in Katowice, Medykow 18 Str, 40-752 Katowice, Poland; 2 Canadian Centre for Health and Safety in Agriculture, College of Medicine, University of Saskatchewan, 104 Clinic Place, P.O. Box 23, Saskatoon, SK S7N 2Z4, Canada; 3 Department of Medicine, College of Medicine, University of Saskatchewan, 103 Hospital Drive, Saskatoon, SK S7N 0W8, Canada; 4 Department of Sports Medicine and Rehabilitation, Yanka Kupala State University of Grodno, 22 Ozheshko Str, 230023 Grodno, Belarus; 5 Department of General and Oncological Pulmonology, Norbert Barlicki Memorial Teaching Hospital No. 1, Medical University of Lodz, Kopcińskiego 22 Str, 90-153 Lodz, Poland; 6 Department of Family Medicine, Warsaw Medical University, Banacha Street 1a, 02-097 Warsaw, Poland; 7 Department of Social Pedagogy and Social Work, Krasnoyarsk State Pedagogical University named after V.P. Astafiev, A. Lebedeva 89 Str, 660017 Krasnoyarsk, Russia; 8 Institute of Biology Systems and Genetic Research, Lithuanian University of Health Sciences, MLK, Eivenių 4 Str, LT-50103 Kaunas, Lithuania; 9 Department of Human Physiology and Pathophysiology, Faculty of Medicine, University of Rzeszów, Kopisto 2a Str, 35-359 Rzeszow, Poland; 10 Department of Allergology and Cystic Fibrosis, Clinical Provincial Hospital No. 2, Lwowska 60 Str, 35-301 Rzeszów, Poland; 11 Department of Epidemiology, Faculty of Public Health, Slovak Medical University in Bratislava, Limbová 14 Str, 833 03 Bratislava, Slovak; 12 2nd Department of Lung Diseases and Tuberculosis, Medical University of Bialystok, Żurawia 14 Str, 15-540 Bialystok, Poland; 13 Department of General Hygiene and Ecology, Grodno State Medical University, Gorkogo 80 Str, 230009 Grodno, Belarus; 14 Department of Childhood Psychology, Krasnoyarsk State Pedagogical University named after V.P. Astafiev, Lebedeva 89 Str, 660017 Krasnoyarsk, Russia; kovalevsky@mail.kspu.ru; 15 Department of Pneumonology, School of Medicine in Katowice, Medical University of Silesia, Ziolowa 45/47, 40-635 Katowice, Poland

**Keywords:** smoking cessation, cigarettes, e-cigarettes, electronic cigarettes, nicotine, students

## Abstract

Our aim is to assess the smoking cessation and vaping cessation activity, including quit attempts and willingness to quit among university students in Central and Eastern Europe, as well as to investigate personal characteristics associated with smoking cessation and vaping cessation attempts. Data were collected by questionnaire which included 46 questions on cigarette and e-cigarette use. Questionnaires were obtained from 14,352 university students (aged 20.9 ± 2.4 years; cooperation rate of 72.2%). For the purposes of this analysis, only data from exclusive cigarette smokers (*n* = 1716), exclusive e-cigarette users (*n* = 129), and dual users (216) were included. Of all cigarette smokers, 51.6% had previously tried to quit smoking and 51.5% declared a willingness to quit cigarette smoking in the near future. Among all e-cigarette users only 13.9% had ever tried to quit using the e-cigarette and 25.2% declared a willingness to give up using e-cigarette in the near future. The majority of the group did not use pharmacotherapy to quit cigarette (87.5%) or e-cigarette (88.9%) use. Our results indicate that while most university students have some desire to quit conventional smoking, those who use e-cigarettes do not have the same desire.

## 1. Introduction

According to the 2017 Eurobarometer 458 survey, 24% of people aged 15 years or over in the European Union (EU) smoked cigarettes daily [1]. The highest prevalence of tobacco use was observed in Central and Eastern Europe (CEE region) [1,2]. Every year about 1.6 million Europeans die because of smoking related-diseases [3]. Tobacco use also burdens the EU economy [3,4]. It is estimated that in 2009, tobacco reduced national wealth in terms of gross domestic product (GDP) by 4.6% of the EU’s GDP [4]. Smoking cessation is a core activity with benefits both for individual smokers and society from a public health perspective [5,6]. Quitting smoking is beneficial to health at any age; however quitting smoking before age 40 years reduces the risk of death associated with continued smoking by about 90% [7,8].

It is estimated that approximately 70% of smokers in Europe want to quit [9,10]. Among current smokers in the EU, 54% had at some point attempted to stop [1]. Nevertheless, only 15% of smokers had attempted to quit smoking in the last 12 months [1]. The highest prevalence of smoking cessation attempts was observed among younger and highly educated adults [11,12]. The most effective method to stop smoking is a combination of cessation pharmacotherapy and behavioural support delivered by healthcare professionals [6,13,14]. A comprehensive tobacco control policy, including smoking cessation services are proven to decrease the prevalence of tobacco product use [15]. However, an analysis of tobacco control policies in EU Member States revealed gaps in the implementation of the tobacco control across the countries, especially in terms of providing comprehensive cessation service [5,11,16].

In recent years, electronic cigarettes (e-cigarettes) were marketed as an effective smoking cessation tool [17,18,19]. Between 2012 and 2017 the proportion of current tobacco smokers in the EU who had ever tried to quit tobacco smoking using e-cigarettes increased from 7.1% to 15.6% respectively [5]. However, there is a scientific debate around whether e-cigarette use is effective as a tobacco smoking cessation method [17,20,21,22]. In addition to this, there are few studies investigating the cessation of vaping, with inconsistent results [23,24,25,26,27]. Data on vaping cessation may be crucial to fully understand vaping behaviours, especially among smokers who switched from cigarettes to e-cigarettes and may inform whether vaping is a next step to end nicotine addiction or rather permanent replacement. In some cases, conventional smokers may use vaping as a way to quit conventional smoking. However, it is still important to study vaping cessation as evidence around the use of e-cigarettes as a conventional smoking cessation method is inconclusive [23,24,25,26,27].

Because of the high burden of tobacco use in CEE [1,2] and the growing popularity of e-cigarettes [28,29,30] especially among adolescents and young adults we aimed to: (1) Estimate the frequency of cessation attempts among smokers and vapers; (2) estimate the frequency of willingness to quit conventional smoking or e-cigarette use among smokers and vapers, respectively; (3) identify personal characteristics associated with cessation attempts and willingness to quit among young adult cigarette smokers and e-cigarette users in Central and Eastern Europe. Conventional smoking products and electronic cigarettes can be used exclusively or in combination and these different user groups may exhibit unique characteristics. As such, we investigated the research questions considering these different combinations as well as general use of conventional and electronic cigarettes. This study is the part of YoUng People E-Smoking Study—YUPESS study [30], including centres from five European countries: Belarus, Lithuania, Poland, Russia and Slovakia.

## 2. Materials and Methods

### 2.1. Study Design and Population

This study is based on a cross-sectional survey carried out between 2017 and 2018, as a part of an international multi-centre research project—YoUng People E-Smoking Study (YUPESS) [30]. The participants were university students from five European countries: Belarus (BY), Lithuania (LT), Poland (PL), Russia (RU) and Slovakia (SK). In each research centre, all students attending school from selected faculties were eligible to be included in the study. The study design and research protocol were described in detail in a previous article [30]. Our study was based on convenience samples. In brief, participants were recruited through universities in each centre. A medical education centre from each location was mandatory. An additional education centre (non-medical) from each centre was also randomly selected for inclusion. Depending on the educational discipline, participants were assigned to either the medical or non-medical student group. All students attending a selected school from selected faculties in the five countries were eligible to be included in the research. Paper-based questionnaires were delivered to each of the subjects personally before the classes by a member of the YUPESS research team.

Completion of the questionnaire was completely anonymous. All the participants had the right to refuse to participate without giving a reason at every stage of the study. The study protocol was reviewed and approved by the Ethical Review Board at the Medical University of Silesia in Katowice, Poland (document number: KNW/0022/KB/205/16).

### 2.2. Study Questionnaire 

The research tool was an original questionnaire developed for the purpose of this study [30]. Questions were included regarding basic socio-demographics, health, and tobacco use. This included 46 questions on cigarette and e-cigarette use. Questions also addressed the personal motives related to cigarette smoking and e-cigarette use as well as behaviours towards smoking cessation and vaping cessation. The covariates were defined by the study protocol [30]. Originally a questionnaire was developed in English and was adapted to the language of each participating country with the use of standard procedures including back-translation. Repeatability of the questionnaire was assessed. In a group of 86 students, an identical questionnaire was conducted twice in an interval of 5–7 days. Kappa coefficients for the critical questions ranged from 0.94 to 1.0. 

Attempts to quit smoking or vaping in the past were based on a positive response to the following: ‘Have you ever tried to quit smoking traditional cigarettes?” and “Have you tried to quit using e-cigarettes (vaping)?’. Intention to quit smoking or vaping in the future was based on a positive response to the following: ‘Are you going to quit smoking in the near future?’ and ‘In the near future you plan to quit using e-cigarettes (vaping)?’. Pharmacotherapy used for smoking cessation was defined based on the question ‘Did you use any of the following pharmaceuticals when quitting smoking of traditional cigarettes?’ with six possible answers as ‘Yes, I used nicotine patches’; ‘Yes, I used nicotine pills’; ‘Yes, I used nicotine gum’; ‘Yes, I used other forms of nicotine-containing products’; ‘Yes, I used non-nicotine products’ and ‘No’. Similarly, pharmacotherapy used for vaping cessation was defined base on the question ‘Did you use any of the following pharmaceuticals when quitting use of electronic cigarettes?’ with six possible answers as ‘Yes, I used nicotine patches’; ‘Yes, I used nicotine pills’; ‘Yes, I used nicotine gum’; ‘Yes, I used other forms of nicotine-containing products’; ‘Yes, I used non-nicotine products’ and ‘No’. Presence of a chronic respiratory condition was based on a positive response to the following: ‘Has a doctor ever said you had any of the following illnesses: Asthma; asthmatic, spastic, or obstructive bronchitis, other chronic respiratory diseases?’.

### 2.3. Statistical Analysis

Statistical analysis was completed using Statistica 12 Software (StatSoft, Tulsa, OK, USA) and SPSS version 25. Initially, descriptive analyses using frequencies (proportions) for categorical variables and means (standard deviations) for continuous variables was conducted. Initial statistical comparisons between groups were made using the independent samples *t*-test or ANOVA for continuous variables or their non-parametric equivalent when appropriate. Statistical testing to compare categorical variables was completed using the independent samples chi-square test. 

Associations between personal characteristics (age, sex, country of residence, presence of a chronic condition) and education group with ever trying to quit smoking or vaping as well as intention to quit smoking or vaping was conducted using multiple logistic regression to adjust for confounders. The strength of association was measured by the odds ratio (OR) and 95% confidence intervals (CI). Statistical inference was based on the criterion *p* < 0.05.

## 3. Results

Questionnaires were obtained from 14,352 students with an overall cooperation rate of 72.2% [30]. For the purposes of this analysis, only data from exclusive cigarette smokers (*n* = 1716), exclusive e-cigarette users (*n* = 129), and dual users (216) were included. The distributions of age, sex, field of study, health status and country of origin for each group of users of nicotine-containing products are presented in Table 1. Among e-cigarette users, 18% used an e-cigarette to quit traditional smoking.

### 3.1. Ever Try to Quit Smoking or Vaping

The proportion of the population who attempted to quit smoking or vaping is presented in Table 2. More than half of all cigarette smokers (51.6%) had previously tried to quit smoking and almost the same percentage (51.5%) declared a willingness to quit cigarette smoking in the near future. Among all e-cigarette users (exclusive e-cigarette users and dual users), only 13.9% had ever tried to quit using the e-cigarette and one-quarter (25.2%) declared a willingness to give up e-cigarette use in the near future. Cigarette smokers made an average of 3 attempts to quit smoking, whereas e-cigarette users averaged only 1.5 attempts to quit an e-cigarette device (Table 2). There were no statistically significant differences in cigarette smoking cessation attempts or willingness to quit between exclusive cigarette smokers and dual users. Moreover, there were no significant differences in e-cigarette cessation attempts or willingness to quit e-cigarette use between exclusive e-cigarette users and dual users (Table 2).

### 3.2. Nicotine Replacement Therapy (NRT) for Smoking Cessation

The majority of the group did not use pharmacotherapy to quit cigarette (87.5%) or e-cigarette (88.9%) use. Dual users were more likely to use pharmacotherapy to quit smoking than exclusive cigarette users (19.7% vs. 11.4%; *p* = 0.001). There was no statistically significant difference (*p* = 0.2) in pharmacotherapy use to quit vaping between exclusive e-cigarette users and dual users. The most popular pharmacotherapy to aid cigarette smoking cessation were nicotine patches (4.5% of all cigarette smokers). Details are presented in Table 3. 

### 3.3. Personal Characteristics Associated with Smoking Cessation Attempts and Plans

The smoking cessation attempts and intentions among cigarette and e-cigarette users by location and personal characteristic are presented in Table 4 and Table 5. The proportion of cigarette smokers who had ever tried to quit smoking significantly differed (*p* = 0.001) across the research Centres with the highest percentage in Slovakia and the lowest in Lithuania. Those with a chronic breathing condition tried to quit cigarette smoking more often compared to those with no respiratory diseases (Table 4). Among all smokers as well as among exclusive cigarette smokers, medical students were more likely to (*p* < 0.05) declare previous attempts to quit or a willingness to quit smoking, compared to non-medical students. There were no statistically significant differences (*p* > 0.05) in previous smoking cessation attempts or willingness to quit smoking among dual users depending on the country of origin, sex, education level or health status (Table 4 and Table 5). 

The proportion of students who had ever tried to quit (Table 4) or planned to quit (Table 5) e-cigarette use (vaping) also differed (*p* < 0.05) between the research centres as presented in Table 4 and Table 5. Among exclusive e-cigarette users, the percentage of students who were willing to quit using e-cigarettes varied from 41.8% in Poland to 5.6% in Russia (*p* = 0.04) (Table 5). Among dual users, the highest percentage of students who had ever tried to quit e-cigarette use was observed in Belarus (27.3%). None of the dual users from Lithuania and Slovakia (*p* = 0.005) declared that they had attempted to quit using e-cigarettes in the past (Table 4).

Results from the adjusted analyses (Table 6) confirmed the descriptive results. Participants from Lithuania and Poland had decreased odds of attempting to quit cigarette smoking compared to Belarus. Participants from Poland had decreased odds of trying to quit using e-cigarettes compared to those from Belarus. Participants from Russia had decreased odds of willing to quit using e-cigarettes in the future (Table 6). Those with a chronic respiratory condition had increased odds of attempting to quit cigarette smoking in the past (Table 6). Non-medical students had decreased odds of planning smoking cessation attempts in the future. Older students had increased odds to have tried to quit cigarette smoking or e-cigarette use in the past and had increased odds of willing to make a smoking cessation attempt in the future (Table 6).

## 4. Discussion

To our best knowledge, this is the first study to examine the smoking cessation and vaping cessation among university students in Central and Eastern Europe. In our study more than half of the smokers had previously tried to quit smoking or planned to quit smoking in the near future. However, relatively few participants (14%) tried to quit e-cigarette using, and only one-quarter of all e-cigarette users declared a willingness to quit using e-cigarette in the near future. Moreover, we did not observe significant differences in the frequency of smoking or vaping cessation attempts and willingness to quit smoking or e-cigarette use between dual users and exclusive cigarette smokers or e-cigarette users. Previous attempts to quit smoking varied between the countries and depended on age of the participants, their health status, and educational background. In our sample, use of pharmacotherapy to support smoking or e-cigarette cessation was generally quite low.

An analysis of smoking cessation trends between 1980 to 2010 in representative samples of the general population from 17 European countries revealed that over three decades, cessation rates increased in all European regions [31]. However, East, South and West European countries were lagging behind North Europe, which suggest the need to intensify tobacco control strategies in these regions [31]. According to the Eurobarometer 2017 survey, the proportion of smokers who had attempted to quit differs across the EU, ranging from 82% in Sweden to 23% in Bulgaria [1]. In our study, the highest percentage of smokers who declared a previous attempt to quit smoking was observed in Slovakia (68.4%). Poland and Lithuania were the countries with the lowest proportion of participants who had ever tried to quit smoking (50.7% and 42.4% respectively). Our results differed from the data on smoking cessation attempts in the general EU population [1]. According to the Eurobarometer 2017 survey, 62% of smokers in Lithuania tried to quit smoking at least once. In the same study, in Poland and Slovakia less than half (48%) of the smokers admitted to previous cessation attempts [1]. The differences in results may be due to the fact that our study was based on a younger population, young adults (students), compared to the general EU population included in the Eurobarometer survey [1]. Smoking cessation attempts may be determined by local tobacco-control policy and smoking cessation services available in each of the participating countries. Findings from the EUREST-PLUS ITC Europe Surveys revealed that the percentage of smokers who receive professional advice to quit varies from 57% in Romania to 21% in Poland [32]. Several studies revealed that physicians in CEE Europe do not routinely identify a patient’s smoking status and do not offer smoking cessation treatment to patients who smoke [32,33,34]. Currently there is a lack of up-to-date data on smoking cessation attempts among smokers from non-EU member countries in Eastern Europe.

We observed that participants with a chronic breathing condition had increased odds of attempting to quit smoking in the past which is in agreement with previously published data [11]. Cigarette smoke may exacerbate the respiratory symptoms, trigger an exacerbation and accelerate the progression of respiratory diseases [35]. Breathing problems and concerns about one’s own health may motivate the patient to quit smoking [35].

In our study, we did not observe differences in the willingness to quit smoking across the countries. However, non-medical students had decreased odds of planning smoking cessation in the future. We can hypothesize that educational background, especially an education in the field of medical and life sciences determines personal behaviours towards smoking, including willingness to quit in the future [30,36]. Also older students had increased odds of trying or willing to quit smoking in the future, which may suggest that with age they were more aware of the harmfulness of smoking and the willingness to quit smoking increased.

Currently, there are only a few studies on vaping cessation attempts among e-cigarette users [23,24,25,26,27]. However, there is no agreement between these studies. The proportion of e-cigarettes users who reported an intention to quit e-cigarette using in the near future varied from 3% in a study by Skerry et al. [24], 8% in study by Etter and Eissenberg [25], 65% in study by Wong et al. [26], up to 73% in study by Ma et al. [27]. In a study by Etter JF [23], 10% of long-term vapers had already tried to quit vaping, and 34% declared an intention to quit vaping in the future. In a study by Canzan et al. e-cigarette use was not associated with smoking cessation in nursing students in Italy [37]. Similar results were observed in our study among university students. In our study 14% of all e-cigarette users had previously tried to quit using e-cigarettes and 25% intended to quit using e-cigarettes in the near future. We did not observe any significant differences between dual users and exclusive e-cigarette users in terms of vaping cessation attempts and intention to stop vaping in the future. Moreover, in our study only 18% of the e-cigarette users reached for an e-cigarette to quit traditional smoking. 

An analysis of smoking cessation assistance in the European Union between 2012 and 2017 showed that the majority of smokers had tried to quit without assistance both in 2012 (70.3%) and in 2017 (74.8%) [5]. Hummel et al. also observed that the majority of attempts to quit smoking in the EU were made without any cessation assistance [32]. In our study only 12% of the participants used pharmacotherapy to aid in quitting smoking and 11% reached for pharmacotherapy in order to quit e-cigarette using. Moreover, we observed that dual users more often used nicotine patches to support smoking cessation compared to exclusive smokers, which may suggest that this group was highly motivated to quit smoking and reached for an e-cigarette as a tool in smoking cessation. The proportion of smokers or e-cigarette users in our study who used pharmacotherapy to quit smoking or vaping was lower compared to the general EU population. Among students, the price of pharmacotherapy used to quit smoking is often a barrier and may result in low usage of NRT and other medications in this group of smokers [38]. National tobacco control strategies should consider access to free-of-charge pharmaceuticals to quit smoking as an element of smoking cessation programs. 

Our study has several limitations. First, our study was based on convenience samples and targeted university students so caution should be taken when trying to generalize the results to the other populations. This is especially true since there are large differences in the study populations and methodologies in the literature. Second, we assessed only current smoking status, which was based on self-reported information provided via paper-based questionnaire. We did not identify ex-smokers or ex-vapers who successfully quit smoking or e-cigarette use. Third, willingness to quit smoking was based on a questionnaire developed by our group. We did not use The Motivation To Stop Scale (MTSS) [39] to assess the personal motivation of the participants to quit smoking. Nevertheless, to our best knowledge, this is the first study to examine the smoking cessation and vaping cessation attempts among university students in Central and Eastern Europe. University students are young people in the risk-taking phase of their lives, and the prevalence of e-cigarette us is especially high in this age group. Moreover, the burden of tobacco use is especially high in the CEE region. Moreover, this study is the first to compare smoking cessation attempts and plans between cigarette and e-cigarette users in the CEE region.

## 5. Conclusions

More than half of the university students in our study had previously tried to quit cigarette smoking with a similar proportion stating that they plan to quit cigarette smoking in the future. A group of university students in Central and Eastern Europe who use e-cigarettes had no intention to quit e-cigarette using, and only a quarter of them intended to quit vaping in the near future. Most smokers or e-cigarette users tried to quit smoking or e-cigarette use without assistance. Medical students and those with chronic respiratory condition were more likely to quit smoking. Older students were more likely to declare willingness to quit smoking or quit using e-cigarette. Previous attempts to quit smoking differed between locations. Our results indicate that while most university students have some desire to quit conventional smoking, those who use e-cigarettes do not have the same desire. There is an urgent need to provide smoking cessation support to all smokers and vapers in Central and Eastern Europe who would like to quit.

## Figures and Tables

**Table 1:** Characteristics of the study population.

	Overall *n* = 2061	Cigarette Smokers *n* = 1716	E-Cigarette Users *n* = 129	Dual Users *n* = 216	*p*
Age, mean (years ± SD)	20.9 ± 2.4	21.0 ± 2.4	20.4 ± 2.2	20.5 ± 2.2	0.001 *
Female (%, 95%CI)	59.8 (57.7–61.9)	62.5 (60.2–64.7)	45.7 (37.4–54.3)	47.2 (40.4–53.6)	<0.001 **
Male (%, 95%CI)	40.2 (38.1–42.3)	37.5 (35.5–39.9)	54.3 (45.7–62.6)	53.0 (46.4–59.6)	
Country					
Belarus	24.5 (22.7–26.4)	23.3 (21.4–25.4)	21.7 (15.5–29.6)	35.6 (29.6–42.2)	<0.001 **
Lithuania	9.6 (8.4–10.9)	9.8 (8.5–11.3)	10.1 (6.0–16.5)	7.4 (4.6–11.7)	
Poland	51.0 (48.8–53.2)	52.4(50.0–54.7)	51.9(43.4–60.4)	39.4(33.1–46.0)	
Russia	10.2 (9.0–11.6)	9.2 (7.9–10.7)	14.0 (9.0–21.0)	15.7 (11.5–21.2)	
Slovakia	4.8 (3.9–5.8)	5.3 (4.3–6.5)	2.3 (0.8–6.6)	1.9 (0.7–4.7)	
Medical students (%, 95%CI)	59.0 (56.9–61.1)	59.4 (57.0–61.7)	59.7 (51.1–67.8)	55.6 (48.9–62.0)	0.6 **
Non-medical students (%, 95%CI)	41.0 (38.9–43.1)	40.6 (38.3–43.0)	40.3 (32.2–48.9)	44.4 (38.0–51.1)	
Have a chronic breathing condition (%, 95%CI)	11.7 (10.4–13.2)	11.6 (10.2–13.2)	10.9(6.6–17.5)	13.0 (9.1–18.1)	0.8 **
Do not have a chronic breathing condition (%, 95%CI)	88.3 (86.8–89.6)	88.4 (86.8–89.8)	89.1 (82.5–93.4)	87.0 (81.9–90.9)	

95%CI—95-percent confidence interval; SD—standard deviation; * result of ANOVA; ** result of Chi-square test.

**Table 2:** Proportion of the population who attempted to quit smoking or vaping and characteristics of those attempts.

	*n*	Attempt to Quit Cigarette Smoking in the Past % (95%CI)	Number of Attempts, (Mean ± SD)	Willingness to Quit Cigarette Smoking in the Near Future % (95%CI)	Attempt to Quit E-Cigarette Using in the Past % (95%CI)	Number of Attempts, (Mean ± SD)	Willingness to Quit E-Cigarette Using in the Near Future % (95%CI)
All cigarette smokers	1932	51.6 (49.4–53.8)	2.9 ± 2.6	51.5 (49.2–53.8)	-	-	-
All e-cigarette users	345	-	-	-	13.9 (10.7–18.0)	1.5 ± 0.6	25.2 (20.9–30.1)
Only cigarette smokers	1716	50.9 (48.5–53.2)	3.0 ± 2.7	51.6 (49.1–54.0)	-	-	-
Only e-cigarette users	129	-	-	-	11.6 (7.2–18.3)	1.5 ± 0.8	31.0 (23.7–39.4)
Dual users	216	57.4 (50.7–63.8)	2.7 ± 2.3	50.5 (43.4–57.7)	15.3 (11.1–20.7)	1.4 ± 0.6	21.8 (16.8–27.7)
*p*		0.07 ^a^	0.3 ^b^	0.8 ^a^	0.3 ^c^	0.9 ^d^	0.06 ^c^

95%CI—95-percent confidence interval; SD—standard deviation; ^a^ result of Chi-square test for exclusive cigarette smokers versus dual users; ^b^ result of Mann-Whitney U-test test for exclusive cigarette smokers versus dual users; ^c^ result of Chi-square test for exclusive e-cigarette users versus dual users; ^d^ result of Mann-Whitney U-test test for exclusive e-cigarette users versus dual users.

**Table 3:** Primary nicotine replacement therapy used by those who attempted to quit smoking or e-cigarette use in the past.

	Pharmacotherapy Used to Quit Cigarette Smoking				Pharmacotherapy Used to Quit E-Cigarette Using			
	All Cigarette Smokers *n* = 934% (95%CI)	Exclusive Cigarette Smokers *n* = 812% (95%CI)	Dual Users *n* = 122% (95%CI)	*p* ^e^	All E-Cigarette Users *n* = 54% (95%CI)	Exclusive E-Cigarette Users *n* = 16% (95%CI)	Dual Users *n* = 38%(95%CI)	*p* ^f^
None	87.5 (85.2–89.4)	88.5 (86.2–90.6)	80.3 (72.4–86.4)	0.001	88.9 (77.8–94.8)	93.8 (71.7–98.9)	86.8 (72.7–94.3)	0.2
Nicotine patches	4.5 (3.3–6.0)	3.6 (2.5–5.1)	10.7 (6.3–17.4)		3.7 (1.0–12.5)	0.0 (0.0–19.4)	5.3 (1.5–17.3)	
Nicotine pills	2.4 (1.6–3.5)	2.7 (1.8–4.1)	0.0 (0.0–3.1)		0.0 (0.0–6.6)	0.0 (0.0–19.4)	0.0 (0.0–9.2)	
Nicotine gum	1.5 (0.9–2.5)	1.2 (0.7–2.3)	3.3 (1.3–8.1)		5.6 (1.9–15.1)	0.0 (0.0–19.4)	7.9 (2.7–20.8)	
Other forms of nicotine containing products	0.9 (0.4–1.7)	0.7 (0.3–1.6)	1.6 (0.5–5.8)		1.9 (0.3–9.8)	6.3 (1.1–28.3)	0.0 (0.0–9.2)	
Non-nicotine products	3.3 (2.4–4.7)	3.2 (2.2–4.7)	4.1 (1.8–9.2)		0.0 (0.0–6.6)	0.0 (0.0–19.4)	0.0 (0.0–9.2)	

95%CI—95-percent confidence interval; *p*—result of Chi-square test; ^e^ Exclusive cigarette smokers vs. Dual users; ^f^ Exclusive e-cigarette users vs. Dual users.

**Table 4:** Smoking cessation and vaping cessation attempts among university students by location and personal characteristics.

	All Cigarette Smokers		Exclusive Cigarette Smokers		Dual Users		All E-Cigarette Users		Exclusive E-Cigarette Users		Dual Users	
	*n*	Ever try to Quit Smoking % (95%CI)	*n*	Ever Try to Quit Smoking % (95%CI)	*n*	Ever Try to Quit Smoking % (95%CI)	*n*	Ever Try to Quit Vaping % (95%CI)	*n*	Ever Try to Quit Vaping % (95%CI)	*n*	Ever Try to Quit Vaping % (95%CI)
Country												
Belarus	477	53.0 (48.6–57.5)	400	52.8 (47.9–57.6)	77	54.5 (43.5–65.2)	105	21.9 (15.1–30.7)	28	7.1 (2.0–22.7)	77	27.3 (18.6–38.1)
Lithuania	184	42.4 (35.5–49.6)	168	42.9(35.6–50.4)	16	37.5 (18.5–61.4)	29	0.0 (0.9–11.7)	13	0.0 (0.0–22.8)	16	0.0 (0.0–19.4)
Poland	984	50.7 (47.6–53.8)	899	50.1 (46.8–53.3)	85	57.6 (47.0–67.6)	152	12.5 (8.2–18.7)	67	16.4 (9.4–27.1)	85	9.4 (4.9–17.5)
Russia	192	53.1 (46.1–60.1)	158	48.7 (41.1–56.5)	34	73.5 (56.9–85.4)	52	9.6 (4.2–20.6)	18	5.6 (1.0–25.8)	34	11.8 (4.7–26.6)
Slovakia	95	68.4 (58.5–76.9)	91	69.2 (59.1–77.8)	4	50.0 (15.0–85.0)	7	14.3 (2.5–51.3)	3	33.3 (6.2–79.2)	4	0.0 (0.0–49.0)
*p*		0.001		0.001		0.2		0.02		0.2		0.005
Sex												
Male	758	52.4 (48.8–55.9)	644	51.7 (47.9–55.6)	114	56.1 (47.0–64.9)	184	14.1 (9.8–19.9)	70	11.4 (5.9–21.0)	114	15.8 (10.2–23.6)
Female	1173	51.1 (48.3–54.0)	1072	50.4 (47.4–53.4)	102	58.8 (56.7–74.8)	161	13.7 (8.7–19.1)	59	11.9 (5.9–22.5)	102	14.7 (8.4–21.9)
*p*		0.6		0.6		0.7		0.8		0.9		0.7
Education												
Medical	1139	53.4 (50.5–56.3)	1019	53.1 (50.0–56.1)	120	55.8 (46.9–64.4)	197	12.2 (8.3–17.5)	77	13.0 (7.2–22.3)	120	11.7 (7.1–18.6)
Non-medical	793	49.1 (45.6–52.5)	697	47.6 (44.0–51.3)	96	59.4 (49.4–68.7)	148	16.2 (11.1–23.0)	52	9.6 (4.2–20.6)	96	19.8 (13.1–28.9)
*p*		0.06		0.03		0.6		0.3		0.6		0.1
Chronic respiratory condition												
Present	227	59.9 (53.4–66.1)	199	59.8 (52.9–66.4)	28	60.7 (42.4–76.4)	42	16.7 (8.3–30.6)	14	14.3 (4.0–39.9)	28	17.9 (7.9–35.6)
Absent	1701	50.4 (48.0–52.8)	1513	49.6 (47.1–52.2)	188	56.9 (49.8–63.8)	302	13.2 (9.9–17.5)	114	10.5 (6.1–17.5)	188	14.9 (10.5–20.7)
*p*		0.007		0.007		0.7		0.5		0.7		0.7

95%CI—95-percent confidence interval; *p*—result of Chi-square test.

**Table 5:** Willingness to quit smoking or vaping among university students by location and personal characteristics.

	All cigarette Smokers		Exclusive Cigarette Smokers		Dual Users		All E-Cigarette Users		Exclusive E-Cigarette Users		Dual Users	
	*n*	Willingness to Quit Smoking % (95%CI)	*n*	Willingness to Quit Smoking % (95%CI)	*n*	Willingness to Quit Smoking % (95%CI)	*n*	Willingness to Quit Vaping % (95%CI)	*n*	Willingness to Quit Vaping % (95%CI)	*n*	Willingness to Quit Vaping % (95%CI)
Country												
Belarus	431	49.9 (45.2–54.6)	368	49.5 (44.4–54.5)	63	52.4 (40.3–64.2)	105	22.9 (15.9–31.8)	28	25.0 (12.7–43.4)	77	22.1 (14.3–32.5)
Lithuania	179	49.2 (41.9–56.4)	163	50.3 (42.7–57.9)	16	37.5 (18.5–61.4)	29	17.2 (7.6–34.5)	13	23.1 (8.2–50.3)	16	12.5 (3.5–36.0)
Poland	945	52.1 (48.9–55.2)	868	52.0 (48.6–55.3)	77	53.3 (42.2–64.0)	152	34.2 (27.1–42.1)	67	41.8 (30.7–53.7)	85	28.2 (19.8–38.6)
Russia	172	50.6 (43.2–58.0)	150	50.7 (42.8–58.6)	22	50.0 (30.7–69.3)	52	9.6 (4.2–20.6)	18	5.6 (1.0–25.8)	34	11.8 (4.7–26.6)
Slovakia	88	59.1 (48.7–68.8)	84	60.7 (50.0–70.5)	4	25.0 (4.5–69.9)	7	14.3 (2.5–51.3)	3	33.3 (6.2–79.2)	4	0.0 (0.0–49.0)
*p*		0.5		0.5		0.7		0.005		0.04		0.2
Sex												
Male	717	52.7 (49.1–56.4)	652	53.1 (49.1–57.0)	95	50.5 (40.7–60.4)	184	27.7 (21.8–34.6)	70	34.3 (24.3–46.0)	114	23.7 (16.8–32.3)
Female	1098	50.6 (47.6–53.5)	1011	50.6 (47.6–53.7)	87	50.0 (39.7–60.3)	161	22.4 (16.1–28.7)	59	27.1 (17.4–39.6)	102	19.6 (12.4–27.5)
*p*		0.4		0.3		0.9		0.2		0.4		0.4
Education												
Medical	1091	55.3 (52.3–58.2)	981	55.5 (52.3–58.5)	110	53.6 (44.4–62.7)	197	26.4 (20.7–33.0)	77	33.8 (24.2–44.9)	120	21.7 (15.2–29.9)
Non–medical	724	45.7 (42.1–49.4)	622	45.7 (41.9–49.5)	72	45.8 (34.8–57.3)	148	23.6 (17.5–31.1)	52	26.9 (16.8–40.3)	96	21.9 (14.8–31.1)
*p*		<0.001		<0.001		0.3		0.6		0.4		0.9
Chronic respiratory condition												
Present	214	54.2 (47.5–60.8)	189	55.0 (47.9–62.0)	25	48.0 (30.0–66.5)	42	28.6 (17.2–43.6)	14	50.0 (26.8–73.2)	28	17.9 (7.9–35.6)
Absent	1597	51.0 (48.5–53.4)	1440	51.0 (48.5–53.6)	157	51.0 (43.2–58.7)	302	24.8 (20.2–29.9)	114	29.9 (21.4–37.9)	188	22.3 (17.0–28.8)
*p*		0.4		0.3		0.8		0.6		0.1		0.6

95%CI—95-percent confidence interval; *p*—result of Chi-square test.

**Table 6:** Adjusted ^g^ associations between smoking cessation outcomes and plans among all cigarette and e-cigarette users by location and personal characteristics.

	Attempt to Quit Cigarette Smoking in the Past OR (95%CI)	Willingness to Quit Cigarette Smoking in the Future OR (95%CI)	Attempt to Quit E-Cigarette Using in the Past OR (95%CI)	Willingness to Quit E-Cigarette Using in the Future OR (95%CI)
Country				
Belarus	1.0	1.0	1.0	1.0
Lithuania	0.59 (0.40–0.87)	0.79 (0.53–1.16)	NA	0.55 (0.17–1.82)
Poland	0.72 (0.56–0.93)	0.79 (0.61–1.03)	0.39 (0.16–0.96)	1.04 (0.50–2.16)
Russia	0.94 (0.66–1.32)	0.95 (0.66–1.36)	0.36 (0.13–1.03)	0.34 (0.12–0.97)
Slovakia	1.29 (0.78–2.14)	0.84 (0.51–1.38)	0.46 (0.05–4.45)	0.29 (0.03–2.70)
Sex				
Female	1.0	1.0	1.0	1.0
Male	0.96 (0.80–1.16)	0.99 (0.82–1.21)	1.03 (0.53–1.99)	1.52 (0.89–2.59)
University				
Medical	1.0	1.0	1.0	1.0
Non-Medical	0.88 (0.71–1.10)	0.73 (0.58–0.92)	1.12 (0.54–2.33)	0.97 (0.53–1.75)
Chronic Breathing Condition				
Absent	1.0	1.0	1.0	1.0
Present	1.56 (1.17–2.07)	1.18 (0.88–1.57)	1.16 (0.44–3.02)	1.16 (0.53–2.52)
Age (years)	1.07 (1.01–1.14)	1.10 (1.03–1.16)	1.08 (0.85–1.37)	1.31 (1.09–1.59)
Year of studies	1.08 (0.99–1.18)	1.05 (0.96–1.15)	1.05 (0.76–1.45)	0.84 (0.65–1.09)

95%CI—95-percent confidence interval; ^g^ Adjusted for each variable in the table; NA: Not applicable as nobody had tried to quit.
